# Editorial: Understanding the heterogeneity and spatial brain environment of neurodegenerative diseases through conventional and future methods

**DOI:** 10.3389/fncel.2023.1211273

**Published:** 2023-05-23

**Authors:** Cui Li, Marissa L. Dubbelaar, Xiaoming Zhang, Jialin C. Zheng

**Affiliations:** ^1^Center for Translational Neurodegeneration and Regenerative Therapy, Shanghai Tongji Hospital Affiliated to Tongji University School of Medicine, Shanghai, China; ^2^Department of Peptide-Based Immunotherapy, University Hospital Tübingen, Tübingen, Germany; ^3^Cluster of Excellence iFIT (EXC2180) “Image-Guided and Functionally Instructed Tumor Therapies”, University of Tübingen, Tübingen, Germany; ^4^Department of Immunology, Institute for Cell Biology, University of Tübingen, Tübingen, Germany; ^5^Quantitative Biology Center (QBiC), University of Tübingen, Tübingen, Germany; ^6^Key Laboratory of Spine and Spinal Cord Injury Repair and Regeneration of Ministry of Education, Orthopedic Department of Tongji Hospital, School of Medicine, Tongji University, Shanghai, China; ^7^Translational Research Institute of Brain and Brain-Like Intelligence, Shanghai Fourth People's Hospital Affiliated to Tongji University School of Medicine, Shanghai, China; ^8^Collaborative Innovation Center for Brain Science, Tongji University, Shanghai, China

**Keywords:** neurodegenerative disease, glia cells, heterogeneity, multi-omics, biomarkers

Neurodegenerative diseases (ND) are characterized by progressive cognitive, psychiatric, and motor deficits due to neuronal loss of the affected brain regions (Cummings and Pillai, 2016). ND usually persist for many years and cause the slow and steady deterioration of one's health, eventually resulting in death. By 2050, the total population over 60 years old are estimated to be 22% (Xu et al., 2017). Here, dementia is one of the leading causes of death and major causes of disability and dependency among the elderly, affecting 55 million people globally according to the World Health Organization and it's expected to surpass 135 million people by 2050 (Collaborators, 2020). As aging is the principal risk factor for many neurodegenerative conditions, the world faces an unprecedented social and economic burden (Ward et al., 2014). For the most common ND, such as Alzheimer's disease (AD), Parkinson's disease, amyotrophic lateral sclerosis, and Huntington's disease, the causes of disease occurrence and progression are not well-known. Moreover, the disease trajectory and severity vary significantly among patients, complicating the diagnosis, study, and treatment. ND present the most significant challenge for modern medicine.

Neurodegenerative disorders, such as AD, are biologically heterogeneous, producing a high variance among *in vivo* disease biomarkers. Current biomarkers includes volumetric measurements from imaging, protein measures from lumbar puncture, or behavioral measurements from psychometrics in disease studies and management. However, the inability to decipher the heterogeneity from the development and progression of the disease over time limits their application for biological insights as well as their utility for patient stratification. One of the key contributors to this heterogeneity is phenotypic heterogeneity meaning that individuals belong to a range of disease subtypes. The other contributor is temporal heterogeneity, including individuals at different stages of a dynamic disease process. Previous studies of biomarker variance typically focus on one aspect of this heterogeneity, either phenotypic heterogeneity at a coarse, typically late disease stage or temporal heterogeneity in a broad population (Hampel et al., 2018). Constructing a comprehensive picture separating phenotypic and temporal heterogeneity would provide insights into underlying disease mechanisms, and enable accurate prognostication, facilitating precision medicine in clinical trials and healthcare.

The heterogeneity and complexity of ND are caused by the interaction of genetic, lifestyle, and environmental factors. The failure of multi-level biological networks and different molecular/cellular alterations create synergy during the disease development (De Jager and Bennett, 2018). The traditional one-size-fits-all diagnostic-therapeutic and clinical research approach must be revised for effective solutions. We need a holistic, systems-level approach to draw a more reliable hypothesis for pathology, which can be accomplished by integrating various layers of data. Omics sciences—genomics, epigenomics, transcriptomics, proteomics, miRNomics, metabolomics, lipidomics, neuroimaging-omics embedded in the systems biology theoretical and computational framework can be used to explain the entire biological continuum of a disease (Li et al., 2018). We can simultaneously identify alterations at different levels, such as transcript, gene, microRNA, protein, lipid, and cellular metabolic processes by analyzing and integrating data derived from varies of parallel technologies. We also can inspect the dynamic molecular alterations under both physiological and pathological conditions at multiple system levels ranging from single subcellular compartments to large-scale biological networks and systems. Therefore, omics science will be crucial to identify novel therapeutic targets and early biomarkers for diagnosis and improve clinical management for patients with complex and heterogeneous ND.

This Research Topic gathers four papers highlighting the potentialities of omics approaches in ND. The proteomics studies characterize all expressed proteins and interacting protein family networks, thus making it a promising method for biomarker discovery. Published manuscripts included two research papers that utilized the proteomics approach to study neurological diseases, one review paper, and one method paper.

Wang et al. investigated the molecular and signaling pathway features during the early phases of seizure events occurring in the hippocampus. In a lithium-pilocarpine-induced acute epileptic rat model, they used a tandem mass tag-based proteomic approach to analyze the global protein expression levels in the hippocampus on day one and day three. Their results suggested that complex macromolecular assembly and cell deaths are modulated occurring on day one and biological protein metabolic processes is modulated on day three.

Liu et al. applied mass spectrometry to identify diagnostic biomarkers for predicting cognitive impairment after aSAH. They applied mass spectrometry to analyze CSF samples from nine patients with impaired cognition and nine patients with intact cognition. They revealed 115 differentially expressed proteins in their discovery cohort. Then they validated NCAM2, NPTXR, NRXN2, RELN, and CNTN2 as biomarkers for cognitive impairment in aSAH patients with an independent discovery cohort.

Simöes Da Gama and Morin-Brureau summarized current human cell models of Blood Brain Barrier (BBB) to study neuropathogenicity related to BBB dysregulation. They examined progress in using human models of the BBB from initial static transwell cultures of brain endothelial cells to models based on microfluidics or organoids derived from human-derived induced pluripotent stem cells through the comparison of the architecture from different model generations as well as the cell types used in their fabrication. They further discussed optimal models to study different types of ND.

Nolle et al. described an immunopanning method to enrich glial cells from human post-mortem tissue for transcriptome and proteome analysis. The immunopanning protocol was employed to acutely isolate microglia, oligodendrocytes and astrocytes from human cortical gray matter tissue and assessed the purity of cells at the RNA and protein levels.

During the development of neurological disease, many cell types intertwined to drive the progression of neuropathology. These interactions dramatically increase the complexity of disease pathology. Moreover, the progressive development of diseases adds another challenging feature. Multi-omics enable us to integrate all layers of information along the longitudinal scale. Nevertheless, Integrating multi-omics data is still a significant challenge in precision medicine with high analytical variance and limitations in experimental design (Grapov et al., 2018). Omics sciences have already shown promising results in describing and predicting the spatio-temporal trajectories of complex multi-factorial diseases such as cancer (Heitzer et al., 2019; Johansson et al., 2019). We can expect the same potential for the application of omics in ND. Clinical researches of ND in big patient cohorts generating huge amount of multi-omic data are the foundation for precision medicine. We can screen for potential biomarkers and druggable targets or identify sub-phenotypes with data-driven multi-omics data analysis in ND. Furthermore, we can understand the pathogenic pathways underlying different disease phenotypes through the integration of different sets of features. Ultimately, we would achieve to define each disease by its biomarkers as a phenotypic subtype with its own suitable personalized treatment.

## Author contributions

All authors listed have made a substantial, direct, and intellectual contribution to the work and approved it for publication.
